# Understanding Outcomes and the Ability to Return to Work After Rotator Cuff Repair in the Workers' Compensation Population

**DOI:** 10.7759/cureus.14213

**Published:** 2021-03-31

**Authors:** Michael J Gutman, Manan S Patel, Akhil Katakam, Nathan Liss, Benjamin M Zmistowski, Mark D Lazarus, John G Horneff

**Affiliations:** 1 Shoulder and Elbow Surgery, Rothman Orthopaedic Institute at Thomas Jefferson University, Philadelphia, USA; 2 Shoulder and Elbow Surgery, Washington University Orthopedics, Saint Louis, USA; 3 Department of Orthopedic Surgery, University of Pennsylvania, Philadelphia, USA

**Keywords:** workers compensation, functional outcomes, rotator cuff repair, laborers, return to work

## Abstract

Introduction

Patients with a worker compensation claim are associated with a greater probability of continued symptoms and activity intolerance. This study aims to determine predictors of improved patient-reported outcomes in the workers’ compensation population.

Methods

Patients with workers’ compensation claims undergoing arthroscopic rotator cuff repair between 2010 and 2015 were included. Age, gender, dominant hand, occupation, and number of tendons involved were analyzed. At a minimum of two years, patients were contacted to complete American Shoulder and Elbow Surgeons (ASES) Survey, Simple Shoulder Test (SST), and return-to-work status (RTW). Preoperative characteristics and scores were then compared.

Results

Seventy patients were available for follow-up at an average of 5.4 years (range: 2.1-8.8 years). Average age was 55 years (range: 37-72); 55 (78.6%) were males, 23 (32.9%) were laborers; and 59 (84.2%) patients returned to work. The sole predictor for RTW was surgery on the non-dominant arm (96.5% versus 75.6%; p = 0.021). Laborers showed decreased RTW (p = 0.03). Patients who completed RTW had excellent outcomes with higher ASES (87 versus 50; p value < 0.001) and SST scores (10.4 versus 4.6; p < 0.001). Patients with three tendon tears had inferior ASES (p = 0.026) and SST (p = 0.023) scores than those with less.

Conclusion

Most workers’ compensation patients have excellent outcomes from rotator cuff repair. Patients with three tendon tear repairs demonstrated the worst functional outcomes. Laborers showed decreased ability to RTW with nearly one-third unable.

## Introduction

In the United States, there are over 4.5 million annual physician visits due to rotator cuff tears [1,2]. Rotator cuff tears are one of the most common forms of upper extremity injury in the workers’ compensation population [3]. Work-related rotator cuff injuries are a common source of limited work productivity and are associated with high costs in compensation claims [3,4]. To date, literature has demonstrated decreased functional outcomes in workers’ compensation patients who underwent rotator cuff tears, total shoulder arthroplasty, and reverse shoulder arthroplasty [5-7]. Although rotator cuff repairs in the workers’ compensation population do have statistically significant improvement after one year following surgery, these patients still show a higher level of disability compared to their non-workers' compensation counterparts [8].

Previous studies have demonstrated significantly worse outcomes in workers’ compensation patients who underwent rotator cuff repair compared to non-workers’ compensation patients who underwent the same procedure [9-11]. Others have compared the characteristics of workers’ compensation patients to those of non-workers’ compensation patients to identify the confounding factors that lead to such worse outcomes [9,10]. Henn et al. reported that even after multivariable analysis controlled for possible confounding factors between the workers’ and non-workers’ compensation populations, worker compensation status independently predicted worse outcomes [12]. The cause of these observed differences between these patient populations is not well understood. Many believe that the worse outcomes demonstrated in the workers’ compensation population can be explained by external issues not related to pathophysiology, such as psychosocial factors or secondary gains like salary or other financial benefits while not in work [9-11]. However, other authors have suggested that fear of reinjury from returning to heavy labor may explain the lower recovery rates [8,13,14]. Although the outcomes of the procedure itself may be good, patients often take longer time to return to work (RTW) after an occupational injury compared to non-occupational injuries [15-17]. This delayed timing of RTW may be a major factor in the perception of poorer outcomes in the workers’ compensation population [14].

Unreliable results can cause hesitation among surgeons in the management of workers’ compensation in patients with rotator cuff pathology. While many previous manuscripts have compared workers’ compensation patients to non-workers’ compensation patients in the rotator cuff repair population, no studies have investigated which factors lead to worsened surgical outcomes within the workers’ compensation population itself. The purpose of this study is to determine predictors of improved patient-reported outcomes in this population as well as the ability to RTW following rotator cuff repair. We hypothesize that patients in this population with larger tears would have worse outcomes and those with “labor-intensive” occupations that require heavy lifting would have lower rates of returning to work.

## Materials and methods

Following institutional review board approval, a query was performed of all patients with rotator cuff tears and billed via workers’ compensation at one institution. The surgeries were performed by one of four fellowship-trained surgeons between January 2010 and December 2015. This query yielded 175 cases in 174 patients. Inclusion criteria required that rotator cuff repairs be performed on patients with workers’ compensation claims and had a minimum follow-up period of two years. Work-related injuries from both acute trauma and repetitive trauma were included. Exclusion criteria were revision of prior rotator cuff tears and patients having undergone any rotator cuff surgery that was not a complete repair. At a minimum of two years, patients were contacted to complete an American Shoulder and Elbow Surgeons (ASES) survey, Simple Shoulder Test (SST), and Visual Analog Scale (VAS) and to provide patient-reported satisfaction score based on a 1-10 Likert scale. Patients were also queried regarding RTW and permanent disability status. In 174 patients meeting inclusion criteria, 70 were available for follow-up at two years. All patients had a magnetic resonance imaging (MRI) scan to determine the number of tendons torn. Operative reports were analyzed to confirm MRI findings and determine number of anchors used in the rotator cuff repair. Chart review was performed on all of these patients to collect age, gender, hand dominance, body mass index, occupation, mechanism of injury, and medical comorbidities including heart disease, hypertension, hyperlipidemia, diabetes, Charlson Comorbidity Index age-adjusted and not age-adjusted, smoking status, and alcohol use. Patients were defined as laborers if they stated that they worked the majority of the time using their shoulders or arms for activities such as lifting (>10 pounds), carrying (>10 pounds), climbing, or repetitive reaching. Patients’ preoperative and intraoperative characteristics and functional scores were compared to assess predictors of ability to RTW.

Postoperative therapy

All patients participated in formal postoperative physical therapy as instructed by our surgeons. Patients wore a sling for six weeks, and formal therapy with a licensed physical therapist was started after six weeks to assist with Phase 1 and Phase 2 stretching followed by progression to Phase 1 and Phase 2 strengthening of the rotator cuff. Strength exercises were delayed until 12 weeks postoperatively. Patients were given progressive lifting restrictions for six months before being allowed to use the arm as tolerated.

Statistics

The data was analyzed by comparing those who returned to work versus those who did not. Continuous data are presented as mean (standard deviation) for parametric data or median (first quartile; third quartile) for nonparametric data. All categorical data are presented as cell count (percent of total count). T-tests were used to calculate p values for parametric data. Mann-Whitney U tests were used for nonparametric data for continuous variables. Chi-square test or Fisher’s exact test was used to calculate p values for all categorical data. Following the univariate, a set of bivariate regressions were analyzed to determine which factors had a relationship with returning to work. Significance was established at p < 0.05. Receiver operator characteristic (ROC) curve analysis was performed on functional scores, and RTW and area under the curve (AUC) values were determined. All statistical analyses were done using RStudio (Version 3.6.1, RStudio, Vienna, Austria).

## Results

Data of 70 workers’ compensation patients who had undergone rotator cuff repair with a minimum two-year follow-up were included in this analysis. The mean follow-up time was 64.9 months (range: 26-105 months) (Table 1). The patients had an average age of 55.1 years (range: 37-72) and were composed of 55 (78.6%) males and 15 (21.4%) females. Out of the 70 patients, 23 (32.9%) were defined as laborers and 47 (67.1%) as non-laborers. Sixty-seven patients (95.7%) reported traumatic injuries rather than repetitive injuries. Forty patients (57.1%), including three ambidextrous patients, experienced injury in their dominant arm (Table 1).

**Table 1:** Patient demographics SD, Standard deviation.

	Total (N = 70)	Unable to Return to Work (N = 11)	Return to Work (N = 59)	P Value
Age (SD)	55.1 (7.2)	52.7 (6.0)	55.5 (7.4)	0.191
Sex				0.233
Female	15 (21.4%)	4 (36.4%)	11 (18.6%)	
Male	55 (78.6%)	7 (63.6%)	48 (81.4%)	
Side of Surgery				1.000
Left	35 (50.0%)	5 (45.5%)	30 (50.8%)	
Right	35 (50.0%)	6 (54.5%)	29 (49.2%)	
Dominant Side				0.021
Non-dominant	29 (41.4%)	1 (9.1%)	28 (47.5%)	
Dominant	41 (58.6%)	10 (90.9%)	31 (52.5%)	
Body Mass Index (SD)	30.6 (5.7)	30.6 (5.0)	30.7 (5.9)	0.988
Laborer				0.032
No	47 (67.1%)	4 (36.4%)	43 (72.9%)	
Yes	23 (32.9%)	7 (63.6%)	16 (27.1%)	
Mental Illness				1.000
No	68 (97.1%)	11 (100%)	57 (96.6%)	
Yes	2 (2.9%)	0 (0.00%)	2 (3.4%)	
Heart Disease				0.602
No	62 (88.6%)	9 (81.8%)	53 (89.8%)	
Yes	8 (11.4%)	2 (18.2%)	6 (10.2%)	
Hypertension				0.387
No	37 (52.9%)	4 (36.4%)	33 (55.9%)	
Yes	33 (47.1%)	7 (63.6%)	26 (44.1%)	
High Cholesterol				0.046
No	45 (64.3%)	4 (36.4%)	41 (69.5%)	
Yes	25 (35.7%)	7 (63.6%)	18 (30.5%)	
Diabetes				1.000
No	56 (80.0%)	9 (81.8%)	47 (79.7%)	
Yes	14 (20.0%)	2 (18.2%)	12 (20.3%)	
Charlson Comorbidity Index				0.740
0	44 (62.9%)	6 (54.5%)	38 (64.4%)	
1	20 (28.6%)	4 (36.4%)	16 (27.1%)	
2	4 (5.7%)	1 (9.09%)	3 (5.1%)	
3	2 (2.9%)	0 (0.00%)	2 (3.4%)	
Charlson Comorbidity Index Age-Adjusted (SD)	1.8 (1.3)	1.4 (1.2)	1.9 (1.3)	0.247

**Figure 1:**
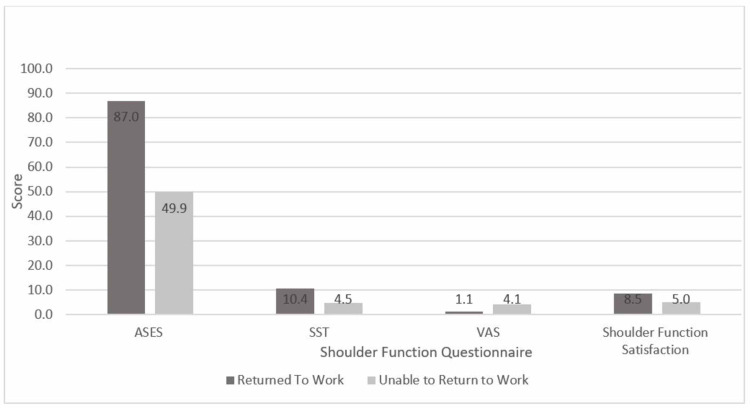
Mean shoulder pain, function, and satisfaction scores ASES, American Shoulder and Elbow Score; SST, Simple Shoulder Test Score; VAS, Visual Analog Scale.

At two-year follow-up, the average satisfaction score with their current shoulder function was 7.9 (range: 0-10), and the mean ASES score was 81.1 (range: 20-100) (Table 2). Fifty-nine patients (84.2%) returned to work. The sole independent predictor of RTW in this population was surgery on the non-dominant arm (96.5% versus 75.6%; p = 0.021). Patients who returned to work had higher final ASES scores (86.9 versus 49.8; p < 0.001), SST scores (10.4 versus 4.54; p < 0.001), and overall satisfaction (8.5 versus 5; p = 0.001). Patients who had lower VAS pain scores had higher rates of returning to work (1.1 versus 4.0; p < 0.001) (Table 2, Figure 1). Laborers showed decreased ability to RTW (p = 0.03) with an incidence of 69.6% (n = 23). However, laborers with non-dominant arm injury compared to laborers with dominant arm injury had RTW rates of 90% (n = 10) and 53.8% (n = 13), respectively (p = 0.09).

**Table 2:** Association between shoulder function and ability to return to work ASES, American Shoulder and Elbow Score; SST, Simple Shoulder Test Score; VAS, Visual Analog Scale.

	Total (N = 70)	Unable to Return to Work (N = 11)	Returned to Work (N = 59)	P Value
ASES	81.1 ± 22.4	49.9 ± 22.2	87.0 ± 17.0	p < 0.001
SST	9.5 ± 3.1	4.5 ± 2.7	10.4 ± 2.1	p < 0.001
VAS	1.6 ± 2.5	4.1 ± 2.5	1.1 ± 2.2	p < 0.001
Satisfaction With Shoulder Function	7.9 ± 2.7	5.0 ± 2.7	8.5 ± 2.4	p < 0.001

ROC curve analysis was performed on functional scores and satisfaction to assess whether there were any cut-off values that were predictive of RTW. Excellent predictive capabilities were found for SST (AUC = 0.937), and good predictive tests were found for ASES score (AUC = 0.896) (Table 3).

**Table 3:** Receiver operating characteristics curve analysis to assess cut-off values to predict return to work AUC, Area Under the Curve; ASES, American Shoulder and Elbow Score; SST, Simple Shoulder Test Score.

Score	AUC	Cut-off 1	Sensitivity	Specificity	Cut-off 2	Sensitivity	Specificity
ASES	0.90	54.17	89.8%	72.7%	84.2	25.4%	90.9%
SST	0.94	6.0	94.1%	77.2%	7.0	93.3%	86.3%
Satisfaction	0.84	7.5	83.0%	81.8%	8.5	67.8%	90.9%

The number of tendons torn ranged from one to three tendons. Twenty-eight patients had one tendon torn, 25 had two tendons torn, and 17 had three tendons torn. Patients with three tendons torn had inferior ASES (p = 0.026), SST (p = 0.023), VAS (p = 0.056), and shoulder satisfaction scores after surgery (p = 0.042) compared to all other patients (Table 4). The number of tendons torn was not associated with the ability to go back to work (p = 0.12). In the 11 patients who did not RTW, four patients underwent a postoperative MRI. In these patients, two were shown to have a recurrence of their tear.

**Table 4:** Functional outcomes based on rotator cuff size ASES, American Shoulder and Elbow Score; SST, Simple Shoulder Test Score; VAS, Visual Analog Scale.

	1 Tendon Tear (N = 28)	2 Tendon Tear (N = 25)	3 Tendon Tear (N = 17)	P Value
ASES	78.6 + 26.0	89.1 + 15.2	73.6 + 22.3	0.026
SST	8.8 + 3.7	10.9 + 1.5	8.5 + 3.0	0.023
VAS	1.82 + 2.7	1.1 + 2.6	1.94 + 1.9	0.059
Satisfaction With Shoulder Function	7.9 + 2.9	8.6 + 2.4	6.9 + 2.8	0.042

There was no statistically significant association with age (p = 0.2), gender (p = 0.2), and body mass index (p = 0.8) with ability to RTW. Medical comorbidities were analyzed to assess their impact on rotator cuff healing affecting their RTW (Table 1). Heart disease (p = 0.6), hypertension (p = 0.4), diabetes (p = 1.00), mental illness (p = 1.00), Charlson Comorbidity Index not age-adjusted (p = 0.74), Charlson Comorbidity Index age-adjusted (p = 0.2), smoking (p = 0.3), and alcohol use (p = 0.6) were not associated with decreased ability to RTW. However, patients with elevated cholesterol were less likely to RTW (p = 0.05).

## Discussion

A number of published manuscripts have found that outcomes of rotator cuff repair in workers’ compensation are worse than those in the non-workers’ compensation population [5,8,11,12]. To our knowledge, no study has solely analyzed patients within the workers’ compensation rotator cuff repair population to determine which factors within this population can be used to predict superior functional outcomes and ability to RTW after rotator cuff repair.

In our study, we found that patients who did not RTW had a higher frequency of rotator cuff tear in the dominant arm. This was especially evident when looking at patients who held labor-intensive occupations that required use of their upper extremities. In general, laborers were less likely to return to work than non-laborers (p = 0.032), but when they had sustained a dominant arm injury, the ability to RTW was decreased even further in 90% of laborers with non-dominant cuff tears able to return to work compared to only 54% of laborers with dominant arm cuff tears. Other authors have attributed this decline in RTW to the inability to meet the demands that a labor-intensive job requires or to psychological factors beyond the loss of shoulder function such as fear of reinjury [8,13,14]. Previous studies have demonstrated that the workers’ compensation population has decreased shoulder function and increased pain after rotator cuff repair surgery compared to the non-workers’ compensation patients [10,11,13,16,18].

As demonstrated in our study, the majority of patients achieved excellent functional outcomes and the ability to RTW. Our authors acknowledge that while other studies demonstrate workers’ compensation has inferior outcomes compared to non-workers' compensation on a population level, workers’ compensation patients on the individual level have the ability to achieve excellent outcomes [8,12]. Our study demonstrates that patients who were able to RTW had significantly improved shoulder pain, functional scores, and surgical satisfaction than those unable to RTW. Moshe et al. showed that in patients with upper extremity disorders, DASH score was the “only independent predictor of RTW” [19]. Likewise, in our cohort composed entirely of workers’ compensation patients, shoulder function and shoulder pain scores were highly predictive of ability to RTW. Patients who returned to work had significantly higher shoulder satisfaction and shoulder function, assessed via ASES and SST surveys, as well as lower pain scores, assessed via the VAS survey. Therefore, within the workers’ compensation population, current shoulder function and pain can be used as predictors of patients’ ability to RTW. As seen by the ROC analysis, cut-offs of 54.2 and 6.0 for ASES and SST are predictive with sensitivities of approximately 90% or greater for RTW. Hence, monitoring postoperative functional progression via these scores can be an effective means to evaluate how close a patient is to returning to work. The correlation of increased functional limitations with being unable to RTW may be either a reflection of severe functional limitation, perceived functional limitation by the patient, the presence of pain interfering with the patients’ activities of daily living, or possibly the result of litigation causing patients’ surveys to be adversely affected. Further studies are warranted to clarify this.

Three tendon tears have been shown to have the highest rate of re-tear rate and worse functional outcomes [20,21]. In the current study, while size of tear (p = 0.12) was not predictive of ability to RTW, patients with three torn tendons experienced the lowest rates of returning to work and the worst functional outcomes. Interestingly patients with two tendon tears experienced better functional outcomes than one tendon tears. One would expect the opposite, given that a larger tear size is typically correlated with worse outcomes. One possible reason for this anomaly in our study could be attributed to the small number of patients. While full-thickness tears can be managed nonoperatively, it is ideal to repair the tendon to prevent further tear extension and muscle atrophy [22,23]. As such, the authors still recommend repair of full-thickness tears regardless of size.

Several limitations exist in this study. This is a retrospective study, which confers to its limitations associated with accurate identification of pathology along with uniform data collection at the time of surgery and preoperative function. Also, factors such as atrophy and fatty degeneration can affect rotator cuff healing and function, which were not assessed in this study. Additionally, although all patients completed extensive postoperative surveys, our study does not have preoperative shoulder functional surveys to assess baseline shoulder function prior to surgery. Ideally, job satisfaction would have been assessed prospectively and preoperatively. This would have allowed our authors to better assess the effects of job satisfaction on long-term outcomes of rotator cuff surgery in the workers’ compensation population. However, preoperative surveys of both shoulder function and job satisfaction may not be as accurate in the workers’ compensation population, since compensation involvement can directly affect patient-reported outcomes including pain, depression, and disability [24]. When assessing ability to RTW, we did not inquire if patients returned to work with the same physical demands or if patients returned to a modified level of work. Additionally, in some patients who were unable to RTW, postoperative imaging was not performed to assess the integrity of the rotator cuff repair. In this cohort, our small sample size may have limited our ability to reach statistical significance. Lastly, we did not inquire on the results of patient litigation for disability. Despite the fact that all consented to be part of the study and were informed that the surveys collected would only be used for research purposes and not recorded in the patients’ medical records, results of litigation may have had a role in how patients answered the questionnaires in our survey [25].

## Conclusions

The majority of patients with workers’ compensation claims have excellent outcomes from rotator cuff repair. Those patients that returned to work were more likely to work as non-laborers, had better functional scores and greater satisfaction with their treatment. Patients with three tendon tear repairs demonstrated worse functional outcomes than small full-thickness tendon repairs.
